# Barriers and facilitators to uptake and persistence on prep among key populations in Southern Province, Zambia: a thematic analysis

**DOI:** 10.1186/s12889-024-19152-y

**Published:** 2024-06-17

**Authors:** Kirsten Stoebenau, Godfrey Muchanga, Sacha St-Onge Ahmad, Chiti Bwalya, Mwangala Mwale, Samara Toussaint, Choolwe Maambo, Carson J. Peters, Caitlin Baumhart, Linah K. Mwango, Marie-Claude C. Lavoie, Cassidy W. Claassen

**Affiliations:** 1 University of Maryland College Park, 4200 Valley Drive, College Park, MD 20742-2611 USA; 2 Present Address: Division of Global Health Sciences, Department of Epidemiology and Public Health, University of Maryland School of Medicine, Baltimore, MD USA; 3 Present Address: Maryland Global Initiatives Corporation Zambia, Lusaka, Zambia; 4 Center for International Health, Education, and Biosecurity, University of Maryland School of Medicine, Baltimore, MD USA; 5 Institute of Human Virology, University of Maryland School of Medicine, Baltimore, MD USA; 6 Ciheb Zambia, Lusaka, Zambia; 7 Division of Infectious Diseases, Department of Medicine, University of Maryland School of Medicine, Baltimore, MD USA

**Keywords:** PrEP, Zambia, HIV, Female sex workers, Men who have sex with men, Sero-discordant couples

## Abstract

**Background:**

Especially in high HIV prevalence contexts, such as Zambia, effective biomedical prevention tools are needed for priority populations (PPs), including key populations (KPs), who are at higher risk. HIV pre-exposure prophylaxis (PrEP) has been scaled up nationally in Zambia, but little is known about barriers to PrEP use among specific PPs to date.

**Methods:**

To understand barriers and facilitators to PrEP use in Zambia, we conducted a qualitative case study of PrEP services to PPs including sero-discordant couples (SDCs), female sex workers (FSWs), and men who have sex with men (MSM) in Livingstone. The study conducted in 2021 included in-depth interviews (*n *= 43) guided by the socio-ecological model, and focus group discussions (*n* = 4) with clinic and community-based providers and PrEP-eligible clients including users and non-users across PP groups. We used thematic analysis to analyze data using codes derived both deductively and inductively.

**Results:**

We found multilevel barriers and facilitators to PrEP use. Cross-cutting barriers shared across PP groups included amplifying effects of PrEP being mistaken for antiretroviral drugs used to treat HIV, including anticipated stigma, and concerns about side-effects based on both misinformation and experience. In addition, stigmatized identities, particularly that of MSM, served as a barrier to PrEP use. The fear of being mislabeled as having HIV was of greatest concern for FSWs. Facilitators to PrEP use primarily included the importance of confidential, KP-sensitive services, and the role of informed, supportive family, friends, and peers. Participants across all PP groups urged expanded education efforts to increase awareness of PrEP within the general population toward mitigating concerns of being mislabeled as living with HIV.

**Conclusion:**

To our knowledge, this is the first qualitative study of the PrEP cascade among multiple PPs in Zambia. This study provides important explanation for the low rates of PrEP continuation found in earlier demonstration trials among KPs in Zambia. The study also offers recommendations for programming efforts going forward such as inclusive PrEP awareness campaigns, expanded KP sensitivity training, and related efforts to thwart PrEP stigma while expanding access.

## Background

While global HIV incidence has declined overall, certain priority population (PP) groups, including key populations (KP), remain at higher risk of acquiring HIV [1]. Alongside condoms, biomedical prevention tools, such as pre-exposure prophylaxis (PrEP), may be especially beneficial for PPs, such as sero-discordant couples (SDC), as well as KPs including female sex workers (FSWs), and men who have sex with men (MSM). PrEP is highly effective and holds promise for global efforts to eliminate new HIV infections by 2030 [1]. However, scale-up of oral PrEP has had mixed results; particularly in Sub-Saharan Africa (SSA), which in 2021 accounted for an estimated 59% of new HIV infections globally [2]. Several demonstration trials with PPs have reported low PrEP uptake and even lower levels of persistence on PrEP for three or more months [3–5]. This has also been the case in Zambia, where guidelines for oral PrEP with tenofovir disoproxil fumarate/emtricitabine were first adopted in 2016 [6]. A 2017–2018 pilot implementation study in Lusaka and Livingstone showed relatively high uptake among certain PP and KP groups; however, persistence was low, with only 21% of FSWs and 23% of MSM remaining on PrEP at three months [7, 8].

By 2018, PrEP was scaled-up nationally in Zambia and made available across the country at Ministry of Health facilities, though distribution rates vary across districts and settings. As efforts to broaden access to PrEP continue, in order to build and adapt programs that meet the needs of all PPs it is crucial to understand and address the perceived barriers to uptake and persistence on PrEP, and to identify the factors that may facilitate access.

Studies that have examined the barriers and facilitators to uptake and persistence on PrEP in settings across SSA among PPs including SDCs, MSM, and FSWs, have described complex, multi-level barriers and facilitators at individual, interpersonal, health system, and society levels [9]. Barriers identified at the individual level included concerns about the drug itself, most notably potential side-effects [10–15]; the large size of the pill [16]; and the necessity to take it daily [17, 18]. Studies also reflected concerns that stemmed from a lack of detailed understanding of how PrEP worked, and doubts about its purported efficacy [17, 19, 20]. Barriers identified at the interpersonal level have been specific to intimate partners and include concerns at the intersection of trust, partner-violence, and PrEP use. In studies among the general population, there was concern that PrEP use would be interpreted as indicative of infidelity [17, 18]. There is also a complex relationship between PrEP use and intimate partner violence (IPV). For FSWs in particular, PrEP use may be motivated by past experiences of violence [21]; however, the threat of violence if clients mistook PrEP for antiretroviral drugs (ARVs) for HIV treatment was described as a barrier to its use [12, 22]. With respect to the health system, practical challenges have been raised by PPs including transportation costs, distance to facilities, and time needed to return to clinics for repeat HIV testing and drug refills [10, 16, 18, 19, 23].

Finally, PrEP stigma, a longstanding concern, and barrier, in other settings [24], can come in many forms, most commonly including anticipated stigma (the threat of being stigmatized); enacted stigma (the act of being discriminated against); or internalized stigma (ascribing negative beliefs to self) [25, 26]. While stigma is upheld at the community or society levels, it is experienced across multiple levels. In other contexts, PrEP stigma has manifested as negative stereotypes ascribed to PrEP users including promiscuity, mistaking PrEP users as HIV positive, and associating PrEP exclusively with stigmatized identities (e.g., MSM) – resulting in intersectional stigmas [26]. PrEP stigma was raised in studies among KPs in SSA, but to a more limited extent. These studies found a widespread concern that PrEP would be mistaken for antiretroviral therapy (ART), and PrEP users mistaken as persons living with HIV (PLHIV) [17, 23]. Further, the stigmatization of minoritized identities was emphasized among MSM, for whom criminalization and threats to personal safety are of very real concern. Specifically, MSM described anticipated and enacted stigma within healthcare facilities as potential barriers to PrEP use [19, 27]; emphasized particularly in contexts where homosexuality is actively criminalized [20, 28, 29].

While extensive barriers persist, a number of factors have been identified as facilitating the use of PrEP among PPs. At the individual level, one primary motivation was high self-perceived HIV risk [18, 30]. PrEP offered peace of mind, allayed fears [31], and facilitated sexual freedom [16, 17, 30, 32–34]. In addition, participants noted concerns with condoms as motivating their use of PrEP, including self-reported dislike of condoms among MSM [32, 35]; partner dislike or inconsistent use of condoms [18, 36]; clients’ preference to forgo condoms among sex workers [12]; or fears of condom failure—emphasized most among FSWs [14, 15, 37]. Motivations for PrEP use at the interpersonal level included family-level motivations such as a desire to stay healthy for their children’s benefit among FSWs and SDC [15, 18], or for SDC, to safely conceive children with an HIV-positive partner [18, 38]; as well as partner-level motivations ranging from suspicions of partner infidelity [18] to a desire among SDC to share the burden of preventing HIV with their ART-using spouse [36]. At community and health system levels, MSM emphasized the importance of having MSM-friendly service providers [32, 33] and reliable social networks for information [29]; and described benefitting from peer educators who could be contacted outside of clinic service hours [32].

While the rapidly expanding literature on perceived challenges and facilitators to PrEP use has provided important insights for programmatic work, we continue to understand too little about the perceived barriers to uptake and persistence on PrEP for KPs in Zambia, despite PrEP now being widely available. To date, no qualitative study has been conducted across multiple PPs using PrEP in Zambia. This qualitative case study aims to identify multi-level barriers and facilitators to uptake and persistence on PrEP among PPs in Livingstone, Zambia to inform and strengthen future PrEP programs.

## Methods

### Qualitative study design

We conducted a study drawing from principles of qualitative case studies. We focused our data collection on a single, bounded case – a clinic located in Livingstone, Zambia working with the Zambia Community HIV Epidemic Control for Key Populations (Z-CHECK) project to offer PrEP services to PP including KPs. Our study focuses on data collected from a range of providers and patients associated with the Z-CHECK project at this specific clinic.

We focus on a single case because this study is situated within a broader effort to evaluate Z-CHECK, and this was the only clinic providing PrEP services to KPs using the Z-CHECK model. The clinic was small and inconspicuous, located off a main road. It was comprised of one main building where medical examinations and outpatient procedures occurred and a few much smaller buildings for private consultations that were located behind the clinic.

### Description of Z-CHECK project

The University of Maryland, Baltimore and its partners implemented Z-CHECK from October 2016 to September 2021, with PrEP services beginning in 2017. Z-CHECK was a community-based project that aimed to interrupt HIV transmission in targeted regions by both identifying and linking HIV-infected persons to care, and guiding uninfected, at-risk persons to prevention care. Z-CHECK was one of the first HIV service delivery grants for KPs in Zambia; and focused on shifting services  from health facilities to KP peer community health workers (CHWs), or CHWs who shared identities with the groups they served. The KP CHWs were identified through partnerships built between Z-CHECK implementers and KP community gatekeepers, namely, established civil society organizations (CSOs) who advocated for MSM and sex workers (SWs). Once trained, CHWs worked alongside Z-CHECK staff to conduct KP-specific community mobilization and sensitization to increase awareness and improve knowledge on PrEP, build trust, and link communities with health facilities. In addition, Z-CHECK staff and clinic-based healthcare providers were trained in KP sensitivity, safety, and security. Participating KPs were individually screened using Ministry of Health high-risk HIV screening tools, and those who tested HIV-negative were linked to prevention services including PrEP.

### Recruitment, data collection, and positionality of research team

Data were collected in September 2021 by a small research team of trained Zambian interviewers comprising two men and three women alongside a U.S.-based researcher with experience working with KPs in other settings. The field team had extensive prior qualitative fieldwork experience on studies concerning HIV treatment and prevention, but not with KP groups. To prepare for this study, we held a nine-day training, including a two-day workshop with Z-CHECK program implementers and leaders of two CSOs who support FSWs and MSM, respectively. This workshop served multiple purposes including to build trust between the field team and CSOs; provide anti-bias training and greater understanding of the lived experiences of FSWs and MSM; refine the sample criteria and interview guides with input from the CSOs and program implementers to improve salience; and finalize the process for participant recruitment. The research team collaborated with Z-CHECK and associated CSO leaders to purposively sample PPs receiving PrEP services, as well as program implementers (Table 1).

**Table 1:** Description of sample by methods of data collection for case study

Participant Type	Methods		Total
	Interviews	FGDs	
**PrEP-Eligible Clients**			
Women in sex work (FSWs)	12	2 (*n* = 13)	25
Men who have sex with men (MSM)	12	2 (*n *= 11)	23
Heterosexual sero-discordant couples (SDC)	6		6
**Providers and Stakeholders**			
Community Health Workers (CHWs)			
MSM CHWs	3		3
FSW CHWs	3		3
Facility-based healthcare workers (HCW)	2		2
Civil Society Organization (CSO) Leaders	2		2
** Total Participants**	**40**	**24**	**64**

The recruitment process entailed PrEP clients being contacted through program implementers or CSO leaders and invited to participate in the study. The sample of PrEP clients was stratified by PP type (MSM, FSWs, SDCs) and across the PrEP cascade, including, among those invited to start PrEP: those eligible but who did not initiate, those who discontinued within three months, and those who continued on PrEP for at least three months (Table 2). PrEP clients were eligible for study inclusion if they (1) were adults who identified with one of the intended PP groups, and (2a) had been invited to initiate on PrEP or (2b) had begun PrEP through the case study clinic in the last year.

**Table 2:** PrEP client sample details

PrEP-Eligible Clients	KP Group		
	**FSWs**	**MSM**	**SDC**
**Total Participants (n)**	25	23	6
**Participant sample (n) across PrEP cascade**			
*Eligible, but did not initiate on PrEP*			
In-depth Interview (IDI)	3	3	2
Focus Group Discussion (FGD)	6	7	
* Discontinued in* < *3 months (IDI)*	3	3	3
*Continued PrEP for 3* + *months*			
IDI	6	6	1
FGD	7	4	
Mean Age (years)	27.0	24.7	37.8
**Biological Sex**			
% Male		100%	33.3%
% Female	100%		66.7%
**Education**			
% Primary only	41.6	0.0	33.0
% Some secondary	33.0	8.0	16.7
% Completed secondary or greater	33.0	91.9	50.0

Clients were invited to participate in either in-depth interviews (IDIs) or focus group discussions (FGDs) totaling 54 participants. Program implementers, facility-based healthcare workers (HCW), and CSO leaders were identified and recruited purposively for IDIs (*n* = 10). In keeping with a case study-informed methodology, only those working with the Z-CHECK PrEP project were eligible for inclusion in this study.

Using the three primary languages spoken at the study site (Nyanja, Tonga, and English) to collect data, the research team spent 10 days conducting audio-recorded FGDs and IDIs. Participants were interviewed at either the clinic or a community space, while FGDs were conducted at a CSO safe space or the clinic when not open. The clinic was used for data collection as the research team was able to access it off-hours, the location was known and accessible to study participants, and it offered privacy.

### Data collection tools

IDI and FGD guides with PrEP clients included the experience of being introduced to PrEP; perceived and experienced barriers, and facilitators to PrEP uptake and persistence (when relevant); and solicited recommendations for improvement going forward. The FGD guides focused more on the social norms and beliefs about PrEP. We assessed perceptions around the perceived barriers, challenges and facilitators of PrEP use through a vignette of a fictional character. For the IDI, we asked about personal experience directly. The questions in both guides were informed by a modified socio-ecological model (individual, interpersonal, health system, community and/or societal). For example, we asked about barriers and facilitators to PrEP uptake at the individual, interpersonal, community and health system levels (e.g., After learning about PrEP what do you remember thinking about it, both good and bad thoughts? Have you talked with your friends about it? What did they say? How was your experience at the clinic? For those who chose not to take PrEP—Let’s think of any worries in the community that may have influenced you. Did you worry about your community or what people might say?) IDIs with providers and stakeholders addressed perceived challenges and successes with delivery of services, uptake, and persistence on PrEP among KPs.

### Data analysis

Data were transcribed, translated, checked for completeness and accuracy, and then uploaded to a qualitative software program (*Atlas.ti* version 9) for management and analysis. Data were analyzed using thematic analysis beginning with deductive codes, then supplemented with emergent inductive codes [39]. The lead author developed the initial deductive code list with corresponding operational definitions focusing on multi-level barriers and facilitators to PrEP use, in collaboration with the analysis team composed of researchers based in Zambia (including the fieldwork team) and the U.S. The codebook was also informed by the socio-ecological model. Codes capturing barriers and facilitators to uptake and persistence on PrEP were used to identify how participants described different levels of influence on their adoption or use of PrEP (e.g. PrEP uptake Barriers – interpersonal – partner; PrEP uptake Barriers – individual – medicine).

Following the collective establishment of an initial codebook, analysis began with an inter-rater reliability check: every team member coded the same interview and then met to resolve discrepancies in interpretation and use of codes. The analysis team of coders were each assigned a subset of transcripts, and met weekly to discuss and agree upon codebook updates and corresponding definitions throughout the coding process. After iterative coding was completed, the lead author developed analytic categories at the intersection of related codes that served as the basis for reducing data on key themes, with analyses stratified by KP subgroups and across the PrEP cascade. Co-authors drafted reports which focused on the barriers and facilitators to PrEP uptake and persistence across levels of influence and disaggregated by KP subgroup, and providers and stakeholders; as well as recommendations for addressing identified barriers. These reports served as the foundation for higher level interpretation and identification of core themes.

## Results

**Fig. 1:**
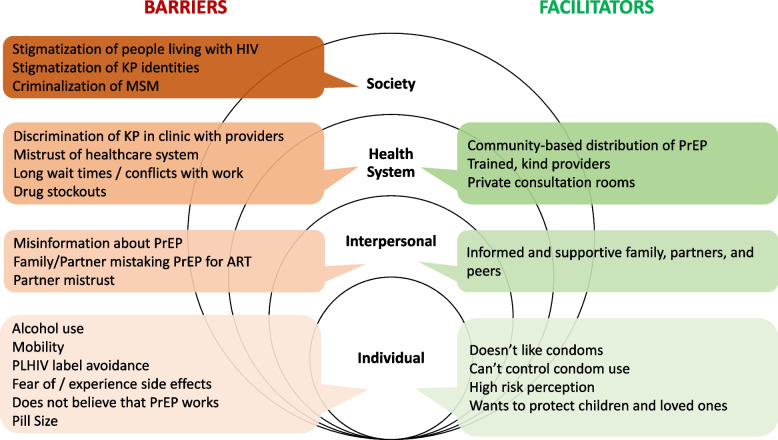
Major facilitators and barriers to PrEP uptake or persistence among PPs in Livingstone Zambia

We present the main results on perceived barriers and facilitators to PrEP use among participants across a modified socio-ecological model (Fig. 1). This includes multiple, and often interdependent, factors at the intrapersonal and individual level; the interpersonal level including partners, family, and peers; the health system level including experiences with providers, clinics, procurement of PrEP, beliefs and trust in the healthcare system; and finally, the broader community and society levels.

### Barriers to uptake and persistence on PrEP

We identified barriers to PrEP across all levels of the socio-ecological model, some emphasized by certain PP groups more than others. We found two themes shared across all groups that cut across levels of influence including (1) anticipated or experienced side-effects, and (2) the amplifying effects of confusing PrEP for ART, some of which were rooted in stigma. We also found that stigmatized identities, especially that of MSM, served as a barrier to PrEP access. Below, we address these key themes, then summarize other barriers across the socio-ecological model.

### Side-effects experienced and anticipated

The experience or threat of side-effects from taking PrEP acted as a barrier to both uptake and persistence for many, and was influenced by interpersonal factors most strongly, though not exclusively. Discussions with family, peers, and friends, particularly those ignorant about PrEP, or themselves wary of it, served to dissuade participants from starting PrEP, or at times convinced them to stop taking PrEP. Further, individual experiences of side-effects hindered some from continuing on PrEP. While these factors were described by all groups, they were emphasized most by FSW.

Participants often recounted the potential negative health effects they heard about from their peers, friends, or family members; most not substantiated by evidence, including organ damage—particularly liver disease, blindness, multiple forms of cancer, and impacts on menstrual cycles. One young FSW who had not initiated on PrEP explained the reasons that people like her might not initiate on PrEP:

*… they get influence[d] from friends who state that the medicines do not work and will just destroy you… they will make you sick with diseases*

Some participants who started PrEP then described becoming convinced by their friends or peers to discontinue. Their friends would recall stopping because they feared serious effects on their bodies and would ask why the participant was still taking PrEP. Health providers also described such discussions and explained that they tried to give eligible patients complete information, including the real potential side-effects, and to dispel myths and misinformation circulating in their networks. One CHW who worked with FSWs explained:

*… we make sure that we give them … full information and they get it from us and no one else. At times you find that maybe a friend would tell them [PrEP did] this and this to me. So …, we make sure that … they [have] full knowledge.* (CHW)

Providers’ described their efforts to provide full information as a means of pushing back against the myths and misconceptions that were prevalent among PP communities.

Alongside these interpersonal influences, some participants described experiencing side-effects that they found too uncomfortable, which they suggested led to their discontinuation. These included vomiting, diarrhea, headaches, dizziness, nightmares, and fatigue. One 35-year-old woman described an overall fatigue she experienced which she explained had led her to stop taking PrEP:

*…the only challenge* *is that the medicine makes me weak, like I have drunk without eating, I don't know. So sometimes you don't feel like taking it. *(FSW, discontinued PrEP)

While many described experiencing one or more of these symptoms, those who continued also noted that they resolved within days or at most two weeks.

In summary, the experience or the anticipation of side-effects served as barriers to uptake or persistence on PrEP for participants across all PP groups.

### The amplifying effect of inadequate differentiation of PrEP for prevention from ART for treatment

Confusion between PrEP and ART was a second multi-level barrier to PrEP use and emerged as a major theme. Participants described their own challenges with understanding the difference between ART and PrEP, but more importantly emphasized their frustration and concern with the limited understanding of PrEP use for prevention within their social networks and the broader community. Participants were concerned about being mistaken for a PLHIV taking ART, reflecting ongoing, entrenched stigmatization of HIV.

At the individual level, some participants expressed concern and disbelief with the extent to which, whether, and in what ways PrEP differed from ART. This stemmed partially from inadequate understanding of the counseling they initially received when being introduced and invited to consider taking PrEP. One 37-year-old woman in a SDC who had initiated and then stopped taking PrEP explained her immediate reaction to learning about PrEP from a provider:

*I was like now this a big lie. They just want us…to contract HIV… the tablets …are the same medicine for HIV.* (IDI with woman in SDC)

Clinic providers and health volunteers within the community also noted this concern. They described clients as reluctant to believe that ARVs could be used for prevention without any health risks. One male CHW who worked with MSM explained that after learning about PrEP, many potential users would simply walk away, saying “*these are just ARVs*.” He went on to explain:

*…it is very common, … you can tell, teach, educate, but somebody is like we have heard but we just don’t want, this … so-called PrEP is the same as ARVs, so don’t cheat us … so, what we do in that [case] is we tell them [we] will come again so that we [can] discuss this more because giving [a] health talk is an ongoing procedure. Today, they will not hear; but tomorrow, they will hear us.* (CHW serving MSM)

For those who were convinced that PrEP could be used effectively for prevention, they then faced addressing ignorance and misinformation within their social networks, including family, friends, peers, and importantly, sexual partners. For some, they explained that this ignorance led them to stop taking PrEP out of fear of being labeled as a PLHIV. As a 21-year-old MSM reflected:

*I was worried that people might be thinking I am taking ARVs because my mom found [my] PrEP and she was surprised, ‘Maybe you are positive.’* *So I got the bottle and threw it in the toilet. They thought I was actually positive.* (IDI with MSM, discontinued)

In other words, because his mother did not know what PrEP was, he remained fearful of being labeled and stigmatized as a PLHIV, and consequently, stopped taking PrEP.

The low levels of understanding about PrEP in the community more broadly also influenced concerns about PrEP clients’ own reputation with friends, peers, and family members. Some MSM suggested that rumors could spread quickly within this tight-knit community, leading to concerns:

*I was worried that if [my friends] hear that I have gone to get PrEP then they will start thinking that I am already sick [HIV positive] and … that is why I am not going for such drugs. (*IDI with 19-year-old MSM, did not start PrEP*)*

The rumor here being a false accusation that he is a PLHIV, again, demonstrating the role of stigma, fueled by poor understanding of PrEP, as a key barrier to both uptake and persistence.

Being identified as a PLHIV could then have additional consequences (e.g., partner violence, being thrown out of living situation, relationships ending), emphasized most by FSWs. In a FGD with FSWs who had not initiated on PrEP they described the concerns they had with PrEP being mistaken for ART, with participants describing the threat of “*rumors in the entire community saying [you are on] ARVs.*” Other consequences were implied in the following exchange:

*I: What do others think might happen if she takes the PrEP medicine home when she stays with her partner?*

*R4: She might be beaten by her partner before she even tries to explain.*

(FGD with FSWs, did not start PrEP)

FSWs were particularly concerned about being mistaken for a PLHIV as this could impact their livelihood; they worried about losing clients. This may explain why concerns about the confusion between PrEP and ARVs featured more heavily among FSWs than other sub-groups. In reference to ignorance about PrEP among her clients, one woman who started PrEP but then discontinued explained:

*Sometimes you ask someone’s status, he tells you he’s negative. He asks you, you also say you’re negative. Now when he sees you drinking that medicine, [he] will think they are ARVs. So that same fear, you’re afraid that you’ll be discovered. Then, they [clients] do not know the …benefits PrEP brings, they do not know.* (35-year-old FSW, discontinued PrEP)

This may also be why FSWs emphasized their hope that broader efforts would be made to educate the public at large on PrEP and the difference between taking the drugs as prevention rather than treatment. One 34-year-old woman underlined how much more could be done by comparing knowledge about PrEP to that of COVID-19:

*Corona has just been there for 2 years, but even young children know about it.* (FSW, continued PrEP*)*

While not emphasized quite as much, other groups shared concern over the limited knowledge about PrEP within the broader community and expressed frustration that the onus of educating the community fell on them.

### Anticipated and enacted stigma among MSM in the clinic and community

Alongside concerns about being mis-identified as PLHIV, MSM in particular also faced extremely high levels of stigma, discrimination, and threats of criminalization attached to their identity as MSM. While all groups shared concerns with accessing care from clinics, and a few FSW also expressed concerns with anticipated stigma attached to their identities, MSM expressed the strongest reservations. The views of MSM held by community members extended into the clinic, where they clienacted stigma among were expressed by fellow patients, as well as the HCWs from whom they hoped to receive care. One MSM serving as a CHW described how a nurse asked MSM awaiting care for their names, and upon hearing them snapped: “*You children with the names from the Bible, what’s making you behave this way?”* In other cases, MSM recounted feeling as though they were put on display, with HCWs not so quietly calling over colleagues to gawk at them. These behaviors were described as hurtful, but worse was the underlying threat of criminalization. In a FGD with MSM, one man recounted a well-known story of a HCW turning a patient over to the police:

*…he went to seek health care services because he had an STI in his [anus]; the clinician called the police on him and that's how he was arrested. So [any other MSM] will obviously be afraid because he will be thinking that the clinicians will call the police on him.* (FGD, MSM, did not start PrEP)

Given the potential threat of criminalization, and despite ongoing efforts to train HCW on how to care for this population, the majority of MSM participants remained wary of seeking healthcare from a clinic. MSM relied on peer recommendations of which clinics were safe and which specific providers could be trusted. As one MSM who had not started on PrEP explained:

*…us KPs, we prefer going to doctors we are familiar with. So if we find any other doctor, we will be skeptical in approaching them because I don't know the response the new doctor will give* (FGD, MSM, did not start PrEP)

This latter point becomes more significant when considering the frequent movement demanded of government HCWs, who are often re-posted. Once a trusted provider leaves, seeking services at the clinic can be perceived as too high a risk to take.

### Additional barriers across the socio-ecological model

In addition to the aforementioned main themes, other factors served as barriers to PrEP use, some of which were more of a concern for certain PP groups as compared to others.

#### Individual-level barriers

At the individual level, MSM and FSW clients, as well as providers, described excessive alcohol use as impacting intended persistence on PrEP. In addition, providers mentioned the mobility of SWs as a barrier for continued PrEP use. Several more practical concerns were commonly raised by many participants, including the size of the pill and challenges with swallowing it (especially among women), forgetting to take the pill every day, or at the same time every day, and concerns about having to take a pill *every* day. Another less common, yet notable, barrier shared by one FSW and one man in a SDC was that using PrEP might result in their increased non-use of condoms, which could increase their risk of other illnesses. A 36-year-old man explained:

*If I am taking [PrEP], then it’s more like I’ll just be encouraging myself to [have] unprotected sex. So let me not go for this.* (man in SDC, did not start PrEP)

In other words, among some of the risk averse, appreciation of the multiple protections afforded from condoms served as a barrier to PrEP use.

#### PrEP and partner dynamics

At the interpersonal level, the use of PrEP could raise issues of intimate partner mistrust. This was particularly, though not exclusively, of concern for MSM in intimate relationships, as the interest in using PrEP signaled either distrust of one’s partner, intentions of infidelity, or both. This is intimated in the following exchange recounted by a 20-year-old MSM CHW describing the challenges faced when introducing PrEP within a relationship:

*‘Babe, I want to start taking PrEP’ then he [asked] me ‘Why do you want to start taking PrEP?’ Then I told him, ‘It protects from HIV.’ Then he said, ‘So you assume that I can give you HIV?’ I told him ‘No.’ Then he [asked] me ‘But why are you taking PrEP if you know I can’t give you HIV*?’ (CHW)

The implication here is that the partner does not understand why taking a drug that protects you from HIV is necessary within the context of a monogamous relationship between two HIV negative members of a couple. It holds a hidden accusation of potential future infidelity. This was emphasized as well in FGDs with MSM. Among MSM FGD participants who had not started using PrEP themselves, a potential partner’s response to their PrEP use was listed as among the most important barriers to adopting PrEP, as one FGD participant indicated:

*The partner will think he's been sleeping around or maybe ‘you don't trust me,’ so I think that would be the most important [reason not to start PrEP*]. (FGD, MSM, did not start PrEP)

For FSWs, by contrast, interpersonal barriers to the use of PrEP with intimate partners were more often rooted in challenges detailed in theme one concerning the confusion between PrEP and ART, as opposed to concerns about infidelity.

#### Pervasive clinic-level barriers to PrEP uptake and persistence

Additional health system-related barriers were shared across all PP groups including long queues, clinic hours conflicting with work schedules, and clinic wait times. The latter sometimes exacerbated concerns that PrEP clients might be confused for PLHIV waiting to receive ARVs, as the lines for prevention and treatment care were not always differentiated. Another barrier mentioned were drug stock-outs, which were particularly acute during COVID-19. Several participants suggested that repeated stock-outs prevented their uptake, let alone continuation, on PrEP.

### Facilitators to uptake and persistence on PrEP

We present facilitators of access to or use of PrEP across the socio-ecological model. We identified four main themes including: (1) perceived HIV risk; and (2) protecting family as individual-level motivations for PrEP use; (3) social support and accountability as interpersonal facilitators; and (4) the importance of welcoming, convenient, and confidential health services at the healthcare and community levels.

### Individual-level facilitators

#### Perceived risk across varying levels of agency

At the individual level, among those who initiated on PrEP, many explained this was motivated by high self-perceived HIV risk resulting from either their sexual behavioral choices, or their constrained capacity to ensure consistent condom use in their sexual relationships.

Alongside the acknowledgement that their work involved having sex with multiple partners, both women and men in SW described pressure from clients to forgo condom use as reasons for their increased HIV risk; and therefore, their interest in using PrEP. Participants in SW also emphasized the difficulty with turning away clients willing to pay more for sex without a condom.

*I think PrEP has worked better because you know when you're a FSW, condoms, some people will say I don't use condoms, or they'll offer some big amount of money. So you say I am using PrEP*, *okay fine. Take the risk, I need that money*. (33-year-old FSW, continued PrEP)

SWs had to balance known risks with immediate financial needs, which caused fear and anxiety that PrEP use helped them to overcome. Several FSWs had also experienced sexual violence with clients or non-partners, such as coerced sex with police officers. The threat of exposure to HIV alongside such violence served for some as an additional motivation to begin using PrEP.

For both MSM and FSWs, the use of PrEP was also motivated by an acknowledgement of one’s own engagement in related risky behavior, including self-described excessive alcohol use impairing their decision-making, as captured by the following 33-year-old FSW:

*…especially when it comes to alcohol. It puts us at a high risk. Now when you drink, you're drunk, no protection. You see, and sometimes the use of condoms, people don't know how to use condoms, it puts them at risk. Even just our behavior …Sometimes when I get drunk, I love beer too much.* (FSW, continued PrEP)

Other participants described their preference for enjoying having multiple sexual partners, while acknowledging this put them at risk. One 30-year-old MSM explained:

*…I am constantly at risk and constantly dependent on PrEP for three years now. So, it has become my life, until the day I will say that I am done with the risky things. (*MSM, continued PrEP)

Finally, participants in SDC also noted that PrEP offered a welcome alternative to condom use.

#### Protecting family

Another motivation for persisting on PrEP, emphasized by many women in SW, and those in SDC, was the interest in ensuring they remained healthy and HIV-free for their children. This is summarized in the following exchange during an interview with a 34-year-old FSW who had continued on PrEP for more than three months:

*I: So your children were the ones that led you to want to be on PrEP?*

*R: Yes [the] thought of their well-being pushed me further … I was like if I get sick then no one will take care of them and they will suffer*. (FSW, continued PrEP)

Similar sentiments were conveyed by those in SDC. A woman who had been in an SDC, but recently separated from her husband, explained how her children had motivated her decision to go on PrEP when she was with him:

*…mmm I just started thinking of my own children that if I don’t take these continuously … then my children will suffer. I was like this disease, if not controlled, can kill you, so that’s how I continued. (*37-year-old woman in SDC, discontinued PrEP*)*

Being motivated to begin PrEP for the sake of one’s children’s wellbeing is indirectly influenced by interpersonal factors. Other interpersonal factors operated more directly, including the role of social support, addressed below.

### Interpersonal facilitators: social support and accountability

While misinformed peers and family were described as discouraging PrEP uptake or persistence; knowledgeable friends, family, and partners were described as key sources of motivation, support, and accountability in persisting on PrEP. Participants across all PP groups indicated that they felt encouraged by friends also taking PrEP, but the role of friends was most apparent among MSM. Many discussed their role in encouraging others to begin PrEP, and how, in turn, these friends or family helped remind them to remain on PrEP. As one 30-year-old MSM who described himself as “a daredevil” because he was the first of his friends to start PrEP explained:

*I introduced a friend of mine… I think I had my close friends, four of them join to [take] PrEP, so we would remind each other.* (MSM, continued PrEP)

Alternatively, FSWs and some in SDC were more likely to mention the role of family members who understood the importance of PrEP as a source of support. After describing her own role in encouraging two sisters and three friends to start PrEP, one 33-year-old FSW went on to talk about the role of her mother:

*…my mother … she's very supportive. She'll even tell me … ‘Even your father could have been alive by this time [if] there was ARVs, PrEP like this’ … She supports me, encourages me, shouts at me, corrects me.* (FSW, continued PrEP)

While not stated directly, this participant’s family appears to have been deeply affected by HIV, and that may be an important source of both knowledge about ARVs and PrEP, as well as motivation to use these drugs effectively for prevention.

### Healthcare system facilitators: welcoming, convenient, and confidential health services

Across all groups, participants emphasized the importance of having reliably welcoming, nonjudgmental, and professional experiences with healthcare providers either at the clinic, or in their community, as significantly influencing their ability to start and continue PrEP. Those who had initiated on PrEP described both interpersonal and structural influences within the health system as important.

The attitude of trained HCWs, including their demonstrated understanding of KP concerns and needs, were especially important to MSM, and many indicated they felt particularly safe at the case study facility, as one 27-year-old MSM who had been on PrEP for more than three months indicated: “*I am comfortable here…I have not seen anyone treat me badly*.”

From a structural perspective, participants emphasized the importance of the establishment of private consultation rooms at the clinic. Unlike many clinics, where services, including pharmaceutical services, are delivered in non-private spaces, the Z-CHECK project created private consultation spaces. One 30-year-old MSM who had continued to take PrEP for more than three months exclaimed:

*You are in a counselling room which has a sign that reads ‘Do Not Disturb’…You actually have more confidentiality there, so there was no one that could know.* (MSM, continued PrEP)

A 34-year-old FSW who had continued on PrEP added:

*…the new method of just going inside the doctor’s room is better because no one will know what you went there for.* (FSW, continued PrEP)

In addition to services at the clinic, some participants had benefitted from services delivered to them in the community. These services included education, health assessment, and community-based drug dispensation. Participants who had benefitted from these services noted their convenience and their perceived safety in receiving services from trained lay providers who were their own peers. As one man, who himself had not yet started PrEP described:

*…we used to fear going to clinics but now health services have been brought close to us … if I have a problem I don't have to go to the facility, I just call them and explain my problem and if it's urgent I go to their place or they come pick and take you to a facility if it's a big problem.* (FGD with MSM, did not start)

As seen in Fig. 1, while factors at the individual level that facilitated PrEP use differed from many of the barriers; at the interpersonal and health system levels, many of the same factors were raised. The conditions and contexts that transformed these factors into facilitators could inform further intervention.

### Recommendations to overcome barriers to PrEP uptake and persistence

Study participants offered numerous recommendations toward either enhancing facilitators or overcoming what they perceived as some of the more important barriers to uptake and persistence on PrEP among their peers or the KPs they served (see Table 3).

**Table 3:** Participant recommendations to address key barriers to uptake and persistence on PrEP

Theme	Sub-theme	Quotes and Related Details	Barriers or Facilitators Addressed
Improve Information and Education	Community: Expand efforts to educate public on PrEP and how it differs from ART	*I really want to see a future where information about PrEP can be on our fingertips. Not whereby you having to follow it to [the] Center. I would like most people in the community to know about PrEP. That's what I want, more information to be given. Not only us but to other people.* (FGD, MSM, did not start)	Anticipatory stigma of misidentification as PLHIV; prevalence of misinformation and myths circulating in community; fears of violence, job loss, and retribution if confused as PLHIV (for sex workers)
	Awareness for Potential PrEP Users	*…we just hear ‘PrEP,’ we want to be taught. We need to hear the side-effects, about this PrEP. Just the knowledge. We just want to know. When you go, don't go for good. … Come back and check on us.* (IDI, 35-year-old FSW, discontinued)	Prevalence of misinformation about PrEP; fears around imagined side-effects
	Instruction	…*you need to explain what it is, how it looks like, and give them samples, they can touch as well. And also tell them exactly what it does in your body. Exactly. So if people don't have that information, they … should really get that scientific explanation.* (IDI, 24-year-old MSM, continued)	Limited knowledge and understanding of PrEP
Address Stigma and Discrimination	Train more providers on how to work with KPs	*So it is very very important [for providers to be able to understand the needs of the MSM community] …to have them trained and have as part of the intensified sensitization training for the PrEP for civil health care service providers and also Community Health Service Providers across the communities so that they can have an absolute understanding and become aware of key population’s existence and of sexual behavior’s existence and also have them of offer services even if they may not necessarily agree.* (CSO leader for MSM)	Stigmatization; discrimination against KPs
	Drug-specific	Differentiate PrEP appearance and packaging from drugs used for ART; Offer injectable PrEP to address partner dynamics and stigma	Anticipatory stigma of misidentification as PLHIV; fears of violence, job loss, and retribution if confused as PLHIV (for FSWs)
Improve and expand PrEP delivery	Delivery of PrEP Services into homes and communities (expand DSD)	*Bring the medication to houses because it’s not everyone that is okay or comfortable with collecting medication from the clinic. Most think that …when/if people see them, they will judge them.* (28-year-old woman in SDC, continued)	Anticipatory stigma of misidentification as PLHIV; fears of violence, job loss, and retribution if confused as PLHIV (for FSWs)
	Increase network connectivity	Expand programs to rural areas; build out infrastructure for informal support groups through social media and DSD platforms to help remind people when to get refills	Enhance social support and accountability
	Pill-specific	Produce smaller pills so it is easier to swallow	Practical barriers to use

Participants strongly recommended expanding information and education on PrEP. Foremost was the insistence that the broader community must be educated on PrEP and how it differed from ART. Participants felt strongly that once the broader community came to understand the mechanisms through which these drugs can be used to prevent HIV, they would hold less fear around collecting and taking them. In addition to educating the broader community, PP participants also emphasized the necessity for repeat follow-up education sessions, so that they did not only “*hear*” about PrEP once but were “*taught*” about PrEP through repeat sessions, including candid discussion of its actual side-effects, preferably from someone who was using PrEP. By working with current PrEP users, myths could be more easily dispelled and real experiences conveyed. Detailed knowledge from a reliable source would also diminish the misinformation circulating in their networks. Further, CHWs and clients suggested modalities for such education including print materials, community drama, large-scale advertisements (e.g., billboards), and workshops in churches and schools.

Another set of recommendations addressed anticipated and enacted stigma against KP members, particularly within the healthcare system. To address concerns raised about how clinics/clinicians and even CHWs introduce and attend to PrEP with KPs, providers and clients recommended expanding sensitivity/educational training to more people, and on additional topics (e.g., mental health, risky sexual practices, quality of care alongside sensitization).

Finally, participants offered numerous recommendations related to the delivery of drugs into the community and the human body. Several participants expressed their wish for an injectable form of PrEP (without necessarily knowing this was under development). Participants described this both as a practical solution to having to take a large pill every day, as well as a means of resolving barriers attached to the concern that PrEP may be confused for ART. It would also alleviate concerns regarding partner trust, as injectable methods would be far more discrete.

Alternatively, participants offered recommendations for differentiating the appearance of ART used for PrEP versus HIV treatment. Suggestions included changing the packaging, or the appearance of the drug itself so as not to be confused with HIV therapy.

Participants also recommended expanding PrEP services into community sites and safe spaces. This recommendation responds to numerous practical concerns with accessing PrEP at a clinic (distance, wait times, opening hours) and, at least as importantly, could address stigma and discrimination. Finally, MSM were most likely to recommend further facilitation of SMS-based support groups, specifically for MSM to share their PrEP experiences, knowledge, and help navigate the process of uptake and persistence. For MSM, feeling safe was incredibly important, and they felt safest when talking with other MSM.

## Discussion

To our knowledge, this case study provides one of the first qualitative assessments of PrEP users in Zambia [40], and is one among a still small, but growing number of studies to focus on PrEP user experiences among KPs in SSA. We identified multi-level barriers and facilitators to PrEP use among different groups of PP participants in Livingstone, Zambia. We found two themes denoting interconnected sets of barriers shared across PP participants regardless of their experience level with PrEP including: (1) side-effects anticipated and/or experienced, and (2) amplified effects of inadequate differentiation of PrEP from ART – with a key subtheme being the anticipated stigma of being identified as a PLHIV. In addition, anticipated and enacted stigma attached to the MSM identity further served to undermine PrEP use among this group. We also identified facilitators of PrEP use at multiple levels of influence, many of which underlined how navigating or overcoming the identified barriers could result in higher levels of uptake or persistence; for example, supportive family and friends who were knowledgeable about PrEP served as important sources of social support and facilitated persistence.

Both concerns about side-effects and confusing PrEP users as PLHIV could be addressed in part through expanded efforts to educate the public, more broadly, on PrEP and what distinguishes PrEP from ARV use for HIV treatment and viral suppression. Similar barriers have been identified in South Africa, Ethiopia, Uganda, and Kenya, where participants also conveyed a reluctance to initiate or continue PrEP due to fears of being wrongly identified as HIV positive [14, 30, 34, 41, 42]. This concern reflects the ongoing depth of the stigma associated with being HIV positive [15], perhaps even more of an issue in contexts where high HIV prevalence means ARVs are easily recognized. Borrowing from the concept of “label avoidance” in stigma research [43]—for example, not seeking mental healthcare to avoid being labeled as mentally ill—forgoing PrEP could be considered “mislabel avoidance,” a means of addressing anticipated stigma of being misidentified as having HIV [44]. To manage mislabel avoidance and enable greater comfort with accessing PrEP in public facilities, storing PrEP in their homes, or being seen taking PrEP, many participants strongly urged efforts to further differentiate the appearance of PrEP from ARVs. It will be important, however, while attending to this concern, to avoid further stigmatizing PLHIV or the use of ART.

The downstream effects of foregoing broader community awareness-raising efforts about PrEP were also apparent in the theme concerning the role of myths and misinformation about PrEP as a barrier to its use. When participants described significant people in their lives having accurate information about PrEP, or as users themselves, these individuals facilitated participants’ uptake or continuation on PrEP; a similar effect was also noted in studies with FSWs in South Africa [23]. However, when such significant individuals had little or no knowledge about PrEP, or instead shared misinformation, they then represented barriers to uptake, and in some cases explained discontinuation, as also found among FSWs in South Africa [23] and MSM in Kenya [34]. Therefore, positive messaging on PrEP is needed at the community level (e.g., through mass communication campaigns) and at the facility level, to introduce and normalize PrEP, while avoiding furthering PrEP stigma in the process (e.g. [44]). Our findings suggest such efforts may help to dismantle two sets of intersecting barriers to uptake or continuation in this study setting.

Other forms of stigma were also apparent in our work, namely associated with minoritized identities. While not entirely absent from discussion among FSWs, concerns related to identity-based stigma were raised by almost every MSM. This is the first qualitative study of PrEP users to include MSM from Zambia, where homosexuality is criminalized. More than any other group, MSM emphasized the importance (and rarity) of finding a trusted healthcare provider. Providers, KP participants, and representatives from civil society organizations representing LGBTQI + rights agreed on the importance of scaling-up provider training on sexual behaviors that may place MSM at risk, how to address MSM-specific health needs, and anti-bias training. As some MSM indicated, there are MSM all over the country, but there are very few trained providers, concentrated only in a few urban centers, who can provide safe and trusted health services. Our findings contribute to a growing literature documenting the challenge for MSM to find accepting and informed healthcare providers, which poses a significant barrier to their effective and continued use of PrEP [15, 19, 20, 27, 28, 34].

In summary, multiple and intersecting forms of stigma were identified across this study. It is possible to reduce HIV-related stigma through intervention [45], and thoughtful recommendations for how to avoid furthering PrEP stigma, in particular, have been offered from lessons learned in other contexts. To date, the focus for programs and providers in Zambia has been to provide targeted counseling and services to those most at risk, without attention to education efforts within the general population. However, this and other studies suggest that the expediency of this approach may be undermined, ultimately, by the negative effects of pervasive ignorance about PrEP within the general population. In response to these types of concerns, others have recommended inclusive approaches to PrEP awareness campaigns. This could help to not only build knowledge about PrEP within the general population, but also to reduce intersectional stigma; as PrEP campaigns targeted solely at communities who are already stigmatized in society can result in further stigmatizing PrEP, as well as those communities [26]. There is opportunity to avoid this outcome in contexts such as Zambia where PrEP continues to remain largely unknown in the general population.

We also found partner-driven barriers to PrEP use among all participant groups, but the emphasis varied across groups. MSM placed greater emphasis on PrEP as a signifier of infidelity, with related concerns of relationship mistrust as a barrier to PrEP uptake or continuation. To our knowledge, we are the first to report this finding among MSM in the SSA context, although this is a well-documented concern in studies with women and men in heterosexual relationships, including in SDCs [17, 18, 46, 47]. By contrast, other studies with MSM have described uncertainty over a partner’s sexual relationships with others as facilitating their PrEP use [30, 34]. Relationship-related barriers served as among the reasons given for recommendations that PrEP be offered as an injectable, so that PrEP could be used with more discretion, a finding noted in other qualitative studies on PrEP preferences among different PP groups in Zambia [40, 48, 49]. That said, some studies have described the benefits of PrEP-use disclosure to intimate partners for adherence and stigma-management [50], though this comes with fear of violence [51]. However, there were also cases in our study where intimate partners served as a source of encouragement and support. This very likely has to do with differences in the expectations and types of relationships – if there are expectations of monogamy, then PrEP is a threat, while if there are expectations of potential casual relationships, PrEP could be a source of comfort.

Finally, staying healthy for one’s children was a theme raised only among women who had persisted on PrEP. PrEP programs may want to consider using the concept of staying healthy for others, including one’s own children, as a motivational tool to encourage initiation and persistence on PrEP for those for whom such messages may be salient, especially given this motivation for PrEP use has been documented elsewhere [18, 23].

There are important limitations to the transferability of our findings. We are reporting on a single case study of clients and providers attached to one clinic where a program actively working to improve KP members’ access to PrEP had been operating since 2017. Yet, we also conducted this work in late 2021, following nearly a year of interruption in community-based delivery of program services due to the COVID-19 pandemic. This had implications for our study methodology, as we were unable to conduct the intended observational data collection in line with our holistic case study approach. Overall, the research team benefitted from the efforts made in the years prior by Z-CHECK program staff to build relationships with CSOs. These relationships enabled access to hidden and marginalized groups. This did, however, result in a smaller sample than intended for SDC, for whom we did not have a CSO partner. We were careful to emphasize the independence of the research team from the program implementers and the voluntary nature of participation. Still, it may be the case that the research team was associated with the program team by participants and could have influenced some of their responses. Furthermore, the barriers described here, particularly among MSM, are almost certainly experienced far more severely for those trying to access services in settings without targeted programs like Z-CHECK in place.

## Conclusion

We have used a socio-ecological model informed framework to highlight the multiple intersecting influences on uptake and persistent use of PrEP among select PPs in Southern Zambia. By doing so, we demonstrate barriers and facilitators to use that expand our understanding beyond individual-level factors and the clinic to highlight concerns that are produced in society and communities including intersecting stigmas and misinformation. Our findings should be used to inform intervention efforts from mass media health education campaigns at the societal level, to stigma reduction and rights focused approaches at the community and health system levels, to culturally-competent delivery of healthcare and the drug itself at interpersonal and individual levels. Our findings highlight the ongoing importance of social influences on health behaviors, and underscore the importance of addressing such influences to ensure the success of biomedical approaches to preventing HIV.

## Data Availability

The datasets generated and/or analyzed during the current study are not publicly available to further protect the confidentiality of participants who identify as MSM in a context where this behavior can be criminalized, but are available from the corresponding author on reasonable request—Kirsten Stoebenau, kstoeben@umd.edu .

## Abbreviations

ART: Ante-retroviral therapy
CHW: Community health worker
CSO: Civil society organization
FGD: Focus group discussion
FSW: Female sex worker(s)
HCW: Healthcare worker
IDI: In-depth interview
KP: Key population
MSM: Men who have sex with men
PLHIV: Persons living with HIV
PP: Priority Population
PrEP: Pre-exposure prophylaxis
SDC: Sero-discordant couple
SW: Sex worker(s)
Z-CHECK: Zambia Community HIV Epidemic Control for Key Populations
